# Supplementary figures and images for: RcTRP5 Transcription Factor Mediates the Molecular Mechanism of Lignin Biosynthesis Regulation in R. chrysanthum against UV-B Stress
Source: Int J Mol Sci. 2024 Aug 24;25(17):9205. doi: 10.3390/ijms25179205 (PMC11395560; doi:10.3390/ijms25179205)

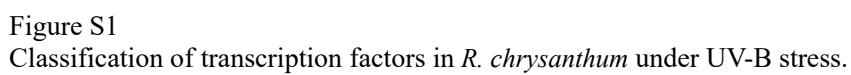

Classification of transcription factors in *R. chrysanthemum* under UV-B stress.

Supplement: Supplementary file 1 [file ijms-25-09205-s001.zip › figure S1.pdf]
